# Functionalized Gold Nanoparticles and Their Biomedical Applications

**DOI:** 10.3390/nano1010031

**Published:** 2011-06-14

**Authors:** Pooja M. Tiwari, Komal Vig, Vida A. Dennis, Shree R. Singh

**Affiliations:** Center for NanoBiotechnology Research, Alabama State University, 1627, Hall Street, Montgomery, AL 36101, USA; E-Mails: poojatiwari@myasu.alasu.edu (P.M.T.); komalvig@alasu.edu (K.V.); vdennis@alasu.edu (V.A.D.)

**Keywords:** gold nanoparticles, functionalization, drug delivery, gene delivery, biosensor, bioimaging

## Abstract

Metal nanoparticles are being extensively used in various biomedical applications due to their small size to volume ratio and extensive thermal stability. Gold nanoparticles (GNPs) are an obvious choice due to their amenability of synthesis and functionalization, less toxicity and ease of detection. The present review focuses on various methods of functionalization of GNPs and their applications in biomedical research. Functionalization facilitates targeted delivery of these nanoparticles to various cell types, bioimaging, gene delivery, drug delivery and other therapeutic and diagnostic applications. This review is an amalgamation of recent advances in the field of functionalization of gold nanoparticles and their potential applications in the field of medicine and biology.

## 1. Introduction

In recent years, the use of nanoparticles, particularly metal nanoparticles has expanded in biomedical research. They are used in diagnosis and therapeutics due to their unique properties of small size, large surface area to volume ratio, high reactivity to the living cells, stability over high temperatures and translocation into the cells, *etc.* They are available in different sizes and shapes due to their ability to react and agglomerate with other nanoparticles in their surroundings. They also exhibit exceptional optical properties making them capable of producing quantum effects suitable for imaging applications. Most commonly studied metal nanoparticles include gold, silver, titanium oxide and iron nanoparticles [1]. Among these, gold being inert and relatively less cytotoxic is extensively used for various applications including drug and gene delivery [2–4]. However, due to their “nano” size, their entry is easily facilitated into various cells posing one of the greatest difficulties in using these nanoparticles for targeted delivery to specific tissues. To obviate this problem, researchers have been conjugating these nanoparticles with various biomolecules and ligands to develop strategies for targeted delivery. Gold nanoparticles (GNPs) and their biomedical applications have been reviewed recently indicating enormous growth in this field [5–8]. The current review focuses on methods of bioconjugation of GNPs, their potential biomedical applications, toxicity and distribution *in vitro* and *in vivo*.

## 2. Synthesis and Functionalization of Gold Nanoparticles (GNPs)

GNPs are the colloidal suspension of gold particles of nanometer sizes. GNPs have been synthesized by an array of methods which mainly are based on the reduction of chloroauric acid in the presence of a stabilizing agent. The most commonly used method, the citrate synthesis method, includes reduction of chloroauric acid using trisodium citrate resulting into the formation of GNPs [2,9]. The size of GNPs is determined mainly by the salt concentration, temperature and rate of addition of reactants resulting in size range of 10–25 nm. However, the size range of 1–100 nm or more can also be achieved by varying the salt concentration and temperature. Another widely used method employs toluene using the tetra-octanyl ammonium bromide as a phase transfer reagent [10]. Several modifications of the basic methods have resulted into an array of techniques to synthesize and manipulate these nanoparticles satiating the needs of a specific research objective [11–13]. Chemical reduction using L-Tryptophane as a reducing agent for ionic gold and polyethylene glycol was used to produce AuCl_4_^−^ ions to provide higher stability and uniformity in size, shape, and particle distribution [14]. Another method used methanol extract of medicinal plants as reducing agent to produce the “GREEN” or environmental friendly GNPs [15]. In another procedure, an amino acid derivative of serrapeptase has been used as stabilizing and reducing agent to synthesize stable “eco-friendly” GNPs [16]. Besides the usual spherical shape, GNPs have been synthesized in various other shapes affecting their physical and biochemical properties. For example, hexagon and boot shaped GNPs show different surface enhanced Raman scattering (SERs) which in turn can be used to detect molecules conjugated to GNPs such as avidin, thereby making these functionalized GNPs (fGNPs) useful for biolabelling, bioassay, clinical diagnosis and therapy [17]. Gold nanocages of six and eight facets have also been synthesized [18]. Similarly, gold nanorods have been synthesized which find usage in biomedical applications for cancer imaging and photothermal therapy [19].

**Figure 1.**
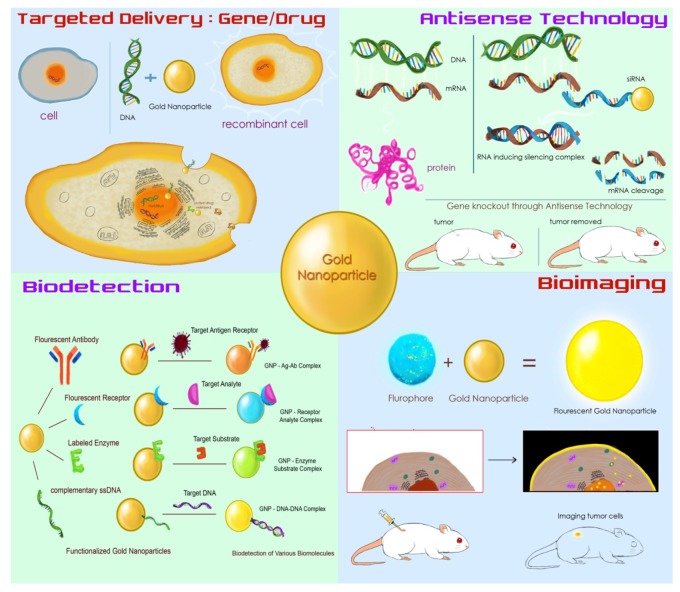
Types of functionalization of gold nanoparticles and their potential biomedical applications.

In yet another recent study, the GNPs were grown in a lysozyme crystal which could be useful as a bifunctional molecule for specific catalytic activity [20]. Methods are being devised to synthesize GNPs with functional moieties (as shown in Table 1) to increase their affinity to biological molecules and use as drug-carriers into the cells with increased specificity. Commonly used functionalization strategies are based on the use of any one or a combination of the groups such as oligo- or polyethylene glycol (PEG), bovine serum albumin (BSA), oligo or polypeptides, oligonucleotides, antisense or sense RNA molecules, antibodies, cell surface receptors and other similar molecules as shown in Figure 1.

### 2.1. PEGylation

PEGylation is one of the most commonly used functionalization methods for GNPs. GNPs are coated with a layer of PEG alone or in conjunction with other molecules such as biotin, peptides or oligonucleotides, thereby helping the internalization of these GNPs to the target cells. Due to their ability to bind the cell membrane, these functionalized GNPs can serve as good drug-carriers. PEGylated GNPs functionalized with biomolecules such as lectin, lactose and biotin have been synthesized [23–25,42–46]. PEGylated GNPs are useful in cellular and intracellular targeting of biological materials. Hetero-bifunctional PEGylated GNPs were synthesized in which GNPs were functionalized with thiol group on one end and coumarin, a fluorescent dye on the other. These fGNPs could make their way into the cells which could be tracked easily because of the attached dye [47]. The stability and functional integrity of PEGylated GNPs is of concern as it is affected by factors such as the molecular weight of PEG, the attached functional groups, the ligand and the size of the GNPs used. The efficacy of one of such group of PEGylated GNPs in the ablation of tumors was tested in mice using thioctic acid anchored PEGylated GNPs [48]. The internalization of these fGNPs was dependent on the size of the nanoparticles, molecular weight of the PEG and the ligands used for PEGylation. Also, the distribution of these GNPs into various cells was dependent on their physiochemical properties.

### 2.2. Peptide/Amino Acid Conjugation

Functionalization of nanoparticles with amino acids and peptides has been another effective way to enhance specificity and efficacy of nanoparticle based delivery systems. GNPs functionalized with amino acids such as lysine, polylysine and glycine bind DNA with higher efficiency for gene delivery without toxicity. Primary ammonium groups of these amino acids contributed to a higher binding capacity to the cationic groups on DNA. Also lysine dendrons were found superior to polylysine for expression of the reporter β-galatosidase gene [49]. Likewise, amine functionalized GNPs carrying siRNA-PEG conjugates against human prostate carcinoma cells were shown to be effective in the inhibition of specific cancer genes [26]. Carboxylated GNPs synthesized using glutamic acid were found better in synthesizing fGNPs with proteins as the carboxyl group of amino acid facilitates attachment of proteins through their amine groups [27]. However, conformational changes were observed in the protein after attachment to the GNPs.

GNPs functionalized with peptides have also been used as effective cell-targeting agents. The peptide CALNN and its derivative CALNNR_8_ were used to functionalize GNPs for targeting intracellular components [28]. Distribution of these fGNPs was dependent on the concentration of the peptide as well as on the size of the GNPs. GNPs (30 nm size) were able to cross cell membrane efficiently by endocytosis and micropinocytosis and showed higher affinity for DNA, RNA and endoplasmic reticulum in the cell. When in mixture both CALNN and its derivative CALNNR_8_ could make their way to the nucleus whereas the CALNNR_8_ was mostly trapped into the endoplasmic reticulum due to the higher affinity of the ER for arginine rich signal peptides. Thus, with constant nanoparticle-diameter and increasing peptide density the cellular targets shift from nucleus to endoplasmic reticulum, whereas when the density of the peptide was kept constant against the diameter of the nanoparticles endocytosis was reduced. The cell viability could be attributed to the extent of fGNP internalization. Similarly, a sensor for the detection of the interaction between β-amyloid peptide with metallic ions Zn^2+^ and Ca^2+^ was designed using GNPs functionalized with β-amyloid peptide-CALNNGK (biotin) G, using standard biotin-streptavidin chemistry [50]. Time dependent study of the interaction between the fGNP was used to suggest the levels of expression of β-amyloid peptide related genes in a simple colorimetric based assay using the optical changes occurring in the absorption spectra of the fGNPs before and after interaction with the peptides.

Peptide functionalized GNPs are also known to activate macrophages, holding promise to be used as adjuvants for vaccine delivery. This is possible due to their ability to bind different biomolecules and expose smaller molecules to the immune system, which are otherwise unrecognizable by the macrophages [30]. The GNPs functionalized with an amyloid growth inhibitory peptide (AGIP) associated with Alzheimer's disease were found useful for intracellular drug delivery. They can selectively target the β-amyloid fibers and sweet arrow peptide (SAP) which could be recognized by the bone marrow derived macrophages. The onset of pro-inflammatory immune response was found to be dependent on the sequence and length of the peptides. However, the macrophages were unable to recognize either of AGIP or SAP alone. These fGNPs were recognized by the macrophages due to TLR-4, a pattern recognition receptor. These fGNPs further activated the pro-inflammatory cytokines TNF-α, IL-1β and IL-6; thus, stopping macrophage proliferation. These fGNPs were then internalized by the macrophages and processed. GNPs can therefore be conjugated to adjuvants, cofactors or adaptor proteins for an effective immune response.

Cellular and subcellular targeting of fGNPs depends on the peptide used for conjugation and the type of cells in question [29]. PEGylated GNPs (30 nm) functionalized with Arg-Gly-Asp (RGD) peptide and a nuclear localization signal peptide lysine-lysine-lysine-arginine-lysine was found to target specifically the nucleus of cancer cells [51]. Likewise, GNPs functionalized with the peptide conantokin-G were internalized by HER293 cells through selective binding to *N*-methyl-D-aspartate receptors [52]. In another study, GNPs functionalized with protein transduction domains (PTDs) from HIV Tat protein were used to follow their intracellular path. PTDs are peptides that can translocate to cell and nuclear membranes in a temperature and receptor independent manner. fGNPs were shown to make their way either into the nucleus (if nuclear localization signal peptide is used) or to the cytoplasm of the target cells through an endosomal path. Peptide sequence thus regulates the entry of the conjugated GNPs. The HIV Tat peptide conjugated GNPs could not enter the HepG2 cells whereas the GNPs with adenoviral nuclear localization signal and integrin binding domain could enter the nucleus.

Peptide-conjugated GNPs are also being used to devise a protein kinase assay using secondary-ion mass spectrometric imaging. This method uses the change in the mass of the peptide substrate after kinase action [53] and is much simpler as opposed to traditional methods using radioactive or fluorescent labels. Thus the peptide conjugated nanoparticles hold promise to be used for bioimaging, diagnosis and therapeutic applications. GNPs are also being functionalized using both peptides and oligonucleotides for perinuclear localization for various functions such as cell imaging, target- specific internalization, *etc.* [54].

Similarly, bioconjugated gold nanorods have been employed as probes for imaging. A mouse monoclonal antibody specific to human epidermal growth factor receptor 2 (HER2), over-expressed in SKBR3 breast carcinoma cells, was conjugated to either GNPs or nanorods which can be used for biomedical imaging of the carcinoma cells [31]. GNPs functionalized with Bombesin peptides, can be used for imaging of cancer cells as Bombesin has high affinity to gastrin releasing peptides that are over-expressed in cancer cells [55]. GNPs coated with polyelectrolytes were found to restructure the 3D constructs made of collagen and cardiac fibroblasts, reduced contraction and altered the expression of β-actin, α-smooth muscle actin and collagen type I, suggesting the potential applications for anti-fibrotic therapies [56]. Likewise, GNPs were also found to enhance cross linking of collagen fibrils as well as sites to deliver signaling compounds that direct self assembly and reduce inflammation [57].

### 2.3. Oligonucleotide Functionalized Nanoparticles

Several research groups have devised methods to functionalize gold and other nanoparticles using oligonucleotides either alone or with some modifications. DNA conjugated nanostructures can be synthesized in a controlled manner, either by attaching a specific number of single stranded DNA molecules through thiol caps or by saturating the surface of the GNPs by single stranded DNA molecules [58]. Kinetic and thermodynamic studies on DNA hybridized to GNPs have shown that ssDNA first adheres to the GNPs and then slowly diffuses on its surface [59]. Secondary structure of a DNA hairpin inhibits interaction between GNPs and DNA thereby increasing the stability of adhered DNA. Aptamer-GNP conjugation has been exploited to target prostate cancer cells [33]. This was achieved by attaching GNPs with an oligonucleotide complementary to the sequence of the anti-PSMA (prostate specific membrane antigen), thus facilitating the attachment of PSMA-GNPs to anti-PSMA antibody. These results show a promising role of such fGNPs in the detection and imaging of cancerous cells.

In another novel study, DNA functionalized GNPs were employed to design a chip based DNA bio bar code sensor to detect target DNA sequences [60]. Here, the bio bar code amplification of the target DNA is assessed using a complementary DNA attached to GNPs and subsequent detection of the amplified DNA instead of the original target DNA.

### 2.4. Other Common Functionalization Methods

Apart from DNA and proteins, various other molecules have also been used effectively for functionalization of GNPs for various applications. GNPs functionalized using goat anti-human IgG were used to formulate a bioassay to detect human IgG in serum samples [61]. GNPs modified with carboxyl and alcoholic groups were functionalized using antibodies for the detection of *E. coli* O157:H7 [62]. GNPs have also been employed in the immobilization of enzymes to offer an inert and biocompatible system [63]. The enzyme glucose oxidase has been immobilized on chitosan-GNPs for the quantitative detection of glucose. This method helps the enzyme retain its activity at higher temperature and extreme conditions. GNPs were used to detect 5-fluorouracil (an anti-leukemic drug) due to the quenching effects of GNPs against the fluorescence of 5-fluorouracil. Also, this conjugate has been shown to have antifungal and antibacterial activity [64].

Apart from these approaches, there have been several studies using different combinations of proteins, DNA, RNA, antibodies, *etc.* to functionalize the GNPs pertaining to the basic requirements of the application in question. The field is expanding day by day and has extensive applications in biomedical sciences.

## 3. Biomedical Applications of fGNPs

fGNPs have found their way from detection to therapeutics in today's medicine based on the functional moieties and their capabilities. GNPs have a significant role in the delivery of nucleic acids, proteins, gene therapy, *in vivo* delivery and targeting, *etc.* Some of the major applications of these fGNPs are listed in Table 1.

### 3.1. fGNPs for Targeted Delivery

fGNPs have been used to target drugs and biomolecules to specific cell types and organelles such as the nucleus or mitochondria. GNPs functionalized with PEG and 3-mercaptopropionic acid was shown to penetrate the nucleus of HeLa cells without causing severe cytotoxicity and hence can be used as a nuclear drug delivery carrier [65]. Similarly, GNPs encapsulated by liposomes have been studied for their cellular targeting and uptake capacity while carrying drugs or other cargos [66]. Intracellular uptake of GNPs as small as 1.4 nm has been shown to enhance internalization by 1000-fold. Such nanoparticles harbor significant potential to be used as gene delivery vehicles, drug-carriers and carriers for other biomolecules.

#### 3.1.1. Gene Delivery

**Figure 2.**
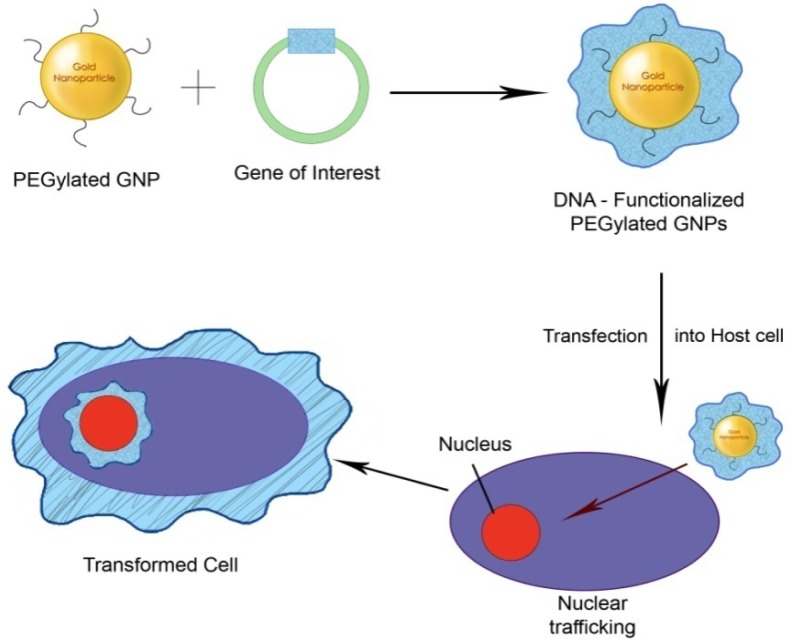
PEGylated gold nanoparticles (GNPs) for gene delivery.

PEGylated GNPs are one of the most commonly used nanoparticles for gene delivery as shown in Figure 2. A PEGylated GNP based delivery system was evaluated for its transfection efficiency using a plasmid DNA mediated through electroporation [67]. Gene expression was enhanced to about 100-fold with DNA-PEGylated GNPs compared to naked DNA after intravenous injection. The transgenes were stable in circulation and the DNA was released and passed through the cellular membranes. Likewise, in another study, plasmid DNA encoding murine interleukin-2 (pVAXmIL-2) was mixed with positively charged colloidal GNPs increasing the transgene expression significantly with reduced toxicity [68]. GNPs functionalized with amino acid have also been used as efficient gene delivery vectors without causing cytotoxicity [49]. Similarly, efficient delivery of siRNA to the host cells was achieved using PEGylated gold-poly (β-amino ester) nanoparticles, wherein the poly (β-amino ester) was the key molecule in the intracellular targeting of the DNA [34].

An adenoviral vector based gene delivery system has been devised by using GNPs immobilized on the magnetic nanoparticles [69]. The problem of viral tropism in the host was avoided along with efficient gene delivery using this technique. These fGNP-DNA conjugates were stable and provided significant transgene expression. Recently, didodecyldimethylammonium bromide (DDAB, a cationic lipid) coated GNPs were reported to offer higher efficiency of gene delivery with reduced toxicity. An increase of more than two-times for green fluorescent protein and 48-fold in luciferase gene expression was observed in cells after transfection [70]. Charge-reversal fGNPs prepared by layer-by-layer technique have been shown to deliver siRNA and plasmid DNA into cancer cells [71]. The Lamin A/C gene, coding for a nuclear envelope protein was achieved by using lamin A/C-siRNA delivered by charge-reversal functional GNPs. This method has a better knockdown efficiency as compared to Lipofectamine 2000. This study signifies the use of such dual purpose functionalized GNPs for targeted delivery as well as gene silencing [71].

Targeted delivery of genes of interest has been achieved successfully using various nanoparticles. In a recent study, DNA functionalized GNPs have been used to modulate gene functions in xenograft tumor bearing mice [72]. fGNPs functionalized with thiolated RNA I could successfully load and deliver antisense DNAs to redirect gene splicing or double-stranded DNAs to decoy gene transcription by transcriptional factors into mammalian cells and *in vivo* mice models, enabling their use in gene delivery and regulation.

Higher transfection effeicency for gene delivery was achived when DNA was conjugated on GNPs complexed with polyethyleneimine (PEI) and chitosan [73]. GNPs functionalized with PEI have been used as a gene delivery vector with reduced cytotoxicity in rabbit cornea [74]. Tissues collected after 12 h, 72 h or 7 days showed appreciable amounts of gold nanoparticles in keratocytes and the extra cellular matrix of rabbit cornea. In another study, GNPs functionalized with PEIs with varying alkyl chain lengths and extent of substitution enhanced the *in vivo* gene expression efficiency in the mouse lung up to 26-fold [75].

#### 3.1.2. Drug Delivery

**Figure 3.**
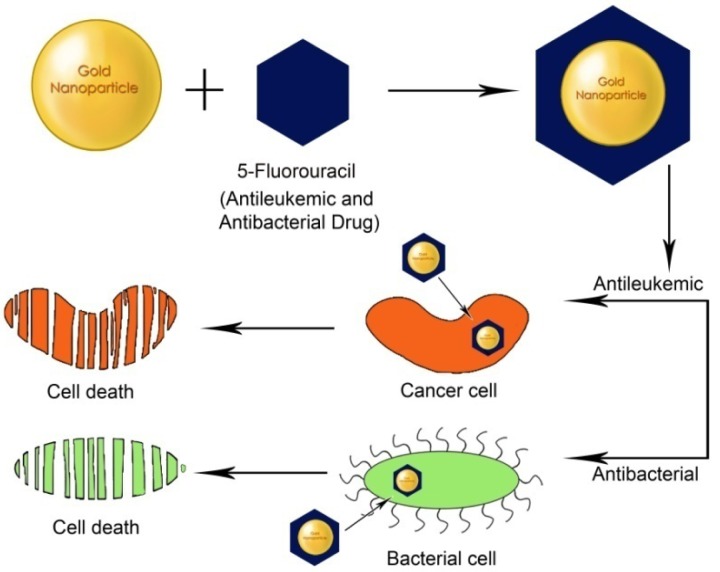
Functionalized GNPs (fGNPs) for drug delivery: Targeting specific cells with higher loading efficiency, targeted delivery and efficient release of drugs.

GNPs are suitable for the delivery of the drugs to cellular destinations due to their ease of synthesis, functionalization and biocompatibility. GNPs functionalized with targeted specific biomolecules can effectively destroy cancer cells or bacteria (Figure 3) [76]. Large surface to volume ratio of GNPs offer a large number of drug molecules being carried by the GNPs [77]. GNPs have been used for the co-administration of protein drugs due to their ability to cross cellular membranes [19], possibly due to the interaction of GNPs with cell surface lipids.

Likewise, GNPs covalently attached to low molecular weight chitosan have been used to design high efficiency vectors for vaccine delivery [78]. These Chito6-GNPs have been studied for their efficiency *in vitro* and *in vivo*. When delivered intramuscularly to BALB/c mice, these conjugates were found more efficient than naked DNA vaccine. Chito6-GNP conjugates also induced potent cytotoxic T lymphocyte responses at a low dose compared to naked DNA.

GNPs functionalized with 5-fluorouracil (5-FU, an anti-leukemic drug) were tested for their antibacterial and antifungal activities against *Micrococcus luteus*, *Staphylococcus aureus*, *Pseudomonas aeruginosa*, *Escherichia coli*, *Aspergillus fumigatus*, and *Aspergillus niger* [64]. 5-FU-GNPs were found to be more effective on Gram negative bacteria than Gram positive due to their easier permeability into the cells. Also, they showed antifungal activity on *A. fumigates* and *A. niger*. However, the mechanism underlying these phenomena remains unclear. In a similar study, GNPs have also been used to detect various aminoglycosidic antibiotics like streptomycin, gentamycin and neomycin [77]. GNPs functionalized with these antibiotics have been evaluated against various strains of Gram positive and Gram negative organisms such as *Staphylococcus aureus*, *Micrococcus luteus*, *E. coli* and *Pseudomonas aeruginosa* suggesting their efficient role in drug delivery. GNPs synthesized using cefaclor as a reducing and capping agent were further encapsulated with PEI and tested for *E. coli* growth. These fGNPs inhibited peptidoglycan layer synthesis of *E. coli*, and increased the cell wall permeability [79].

Gold nanorods functionalized with innate immune response activators were used to inhibit H1N1 influenza virus. Here, ssRNA was used to activate retinoic acid-inducible gene I pathogen recognition pathway which increased the expression of IFN-β and other IFN-stimulated resulting in a decrease in the replication of H1N1 influenza viruses [80].

#### 3.2. As Cancer Diagnostic and Therapeutic Agents

The use of nanoparticles for cancer therapy has been gaining popularity in recent years [81]. GNPs (2 nm) conjugated with cyclodextrin and admantane has shown photothermal effects against cancer cells [82]. The GNPs have also been used in combination with magnetic nanoparticles to target specific cell types for efficient imaging of cancer cells. Nanoparticles can target tumor cells by an accumulation and entrapment process, known as permeation and retention effect imposed by angiogenic vessels and improper lymphatic flow. Therefore, the nanoparticles can accumulate selectively inside the cancerous cells at higher concentrations than the normal cells.

Gold coated iron nanoshells were shown to inhibit the growth of oral and colorectal cancer cells, at a concentration of 5 μg/mL without being toxic to normal cells before oxidation [83]. However, the cytotoxicity was found to be dependent on the age of the nanoparticles. They were found to be released in the human cell lines at slower rate until 48 h and this can be easily detected due to the presence of iron by various biomedical techniques.

GNPs functionalized with fluorescently labeled heparin have been recently used for the targeted detection and apoptotic killing of metastatic cancer cells [84]. The rationale used in the study is the over-expression of heparin-degrading enzymes by metastatic cancer cells. When attached to GNPs fluorescence of heparin is quenched but upon cleavage by heparinase/heparanase the fluorescence is regained and cancer cells can be detected. Also, it was shown that heparin binds to RGD peptide over-expressed in cancer cells inducing apoptosis. These fGNPs could thus be useful for both diagnosis and treatment of cancer. GNPs have been reported to provide a better surface for modifications like PEGylation, Thiol-PEGylation, *etc.* with better colloidal stability and biocompatibility [50]. SH-PEGylated GNPs have been reviewed as a contrast agent for tumor vascular agents. Many type of cancerous cells, such as oral squamous epithelial cells, are difficult to diagnose at an early stage of onset which can be diagnosed using GNPs [85].

GNPs have the ability to exhibit different surface plasmon resonances, when placed close to each other; hence they have been shown to differentiate between the normal and the cancerous cells when conjugated to anti-epidermal growth factor receptor antibodies as a biomarker agent. Gold nanoshells with iron oxide core (Gold-iron oxide nanoshells) have been developed as a promising tool for both tumor cell targeting and treatment due to the ease of preparation, chemical stability, biocompatibility and distinct optical properties [86]. In Gold-iron oxide nanoshells, gold provides optical properties whereas iron oxide core provides magnetic properties for their suitable use in MRI of tumor tissues for diagnosis and therapeutic use in hyperthermic treatment. Although, these nanoparticles have to cross the biological barriers, judicious selection of nanoparticles with respect to size, shape and functionalization method can enhance the guided entry of these nanoparticles into specific tumor targets. Selecting smaller sized nanoparticles can extend the retention time in tumor tissue (passive targeting). Functionalization of such nanoshells using the gold surface through cell surface receptors (e.g., epidermal growth factor receptors, EGFRs), peptides and antibodies against tumor cells can increase the residence of these nanoparticles, thus enabling their use in diagnosis and therapy.

GNPs functionalized with polyamidoamine (PAMAM) dendrimer-folic acid and/or flourescein isothiocyanate (FITC) conjugates have been used for targeting and imaging of the tumor cells [87]. Due to surface properties and acetylation of the terminal amines of these dendrimers, it is possible to synthesize fGNPs with several ligands giving rise to multifunctional nanoparticles. Attachment of folic acid helps these nanoparticles target the tumor cells by binding to the folic acid receptors on the cell membrane *in vitro*. In another similar study, GNPs having a glutathione cap with COOH groups and folic acid in addition to a FITC tag were used to target carcinoma cells [88]. These fGNPs explicitly interacted only with HeLa cells, due to the expression of the folic acid receptors, excluding the non-cancerous cells, thus, providing an easy and sensitive method of cancer cell detection. Also, folic acid attached to PEGylated GNPs has been used to target cancer cells [89]. The PEG backbones used included, PEG diamine, PEG-tetramine, PEG-dithiol *etc.*, for targeted drug delivery. A thiol-PEGylated tamoxifen derivative was developed to selectively target and deliver GNPs to breast cancer cells with up to 2.7-fold enhanced drug potency *in vitro* [90]. PEG-folate GNP conjugates of thioctic acid were used for subcellular targeting of the ovarian cancer cells. These fGNPs could enter various subcellular compartments depending on the drugs/compounds loaded on them. Cisplatin and doxorubicin loaded GNPs targeted nucleus whereas gamitrinibs loaded GNPs targeted mitochondria of the cancer cells [91]. GNPs functionalized with therapeutic agents can be activated through exchange with complementary molecules, thereby reducing cytotoxicity, targeting sub-cellular locations and finally release of the drug for desired therapeutic effect [92]. Similarly, hollow gold nanospheres have been developed with dual capacity of photothermal ablation of cancer cells as well as the release of doxorubicin upon irradiation with near infra red light [93]. Likewise, cylindroid GNPs with fluorescein or doxorubicin have been employed for grug delivery in tumor cells [94]. Porphyran capped gold nanoparticles were used as carriers of anticancer drug (doxorubicin) in human glioma cell line LN-229. The cytotoxicity of this drug was higher when conjugated to GNPs as compared to the drug alone [95].

Biocompatible GNPs with two functional domains have been used for delivery of drugs into cells without being internalized [96]. These domains included a hydrophobic alkanethiol interior and a hydrophilic shell composed of a tetraethylene glycol (TEG) unit terminated with a zwitterionic head group. These functionalized particles minimize non-specific binding with biomacromolecules. It was demonstrated that hydrophobic dyes/drugs can be stably entrapped in hydrophobic pocket of GNPs and released into the cell by membrane-mediated diffusion without uptake of the carrier nanoparticle. The small size of these nanocarriers coupled with their biocompatible surface functionality provides longer circulation lifetime and preferential accumulation in tumor tissues due to the enhanced permeability and retention effect. Additionally, the non-interacting nature of their monolayer makes these systems highly amenable for targeting strategies. B-chronic lymphocytic leukemia (BCLL) is characterized by the increased resistance to apoptosis. GNPs conjugated to anti-VEGF (vesicular endothelial growth factor) have been shown to enhance the induction of apoptosis in CLL cells as compared to these antibodies alone [97].

**Figure 4.**
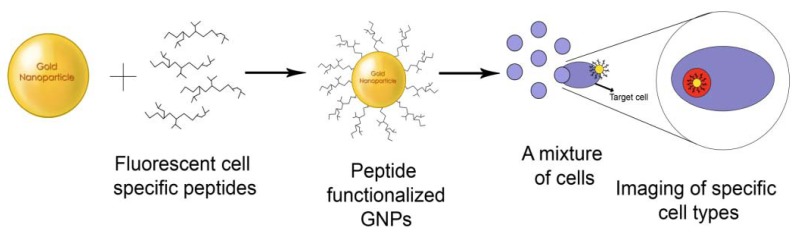
GNPs functionalized with cell specific peptides for bioimaging.

Studies have been conducted on nanoparticles functionalized with fluorophores, peptides, cell adhesion molecules, aptamers or other biomolecules to target specific tissues and thereby holding promise to be used for imaging of tumors, drug delivery and detection of apoptosis [28,55,92,98] (Figure 4).

GNPs functionalized with coumarin and PEG have been shown to be effectively internalized by the human breast carcinoma cells without causing any toxicity [62].This dual functionalization of GNPs involving biomolecules and fluorescent dyes can particularly be used to target cells for bioimaging along with drug delivery purposes. Similarly, GNPs functionalized with octreotide peptide, a synthetic analogue of somatostatin, have been used as potential bioimaging agents for various neuro-endocrine carcinomas [32]. These carcinomas over-express the somatostatin receptors and development of bioimaging agents based on these receptors can help diagnose such tumors. GNPs functionalized with the octreotide were shown to interact more with the tumor cells as compared to GNPs alone, owing to their increased fluorescence properties and enhanced capacity of being recognized by the protein receptors.

Radioactive GNPs functionalized with gum arabic glycoprotein (GA-^198^AuNP) were studied for their biocompatibility and cancer therapeutic applications in severely compromised immuno-deficient (SCID) mice [99]. Individual tumor cells were targeted and nanoparticles were able to penetrate through tumor vasculature and pores with minimum or no radioactivity leakage.

### 3.3. As Biosensors

GNPs have been studied and exploited in the development of an assortment of biosensors to detect specific biomolecules significant in disease etiology. Determination of choline in various human samples is clinically important and is usually assayed through the estimation of the enzyme choline esterase. A biosensor developed by combining choline oxidase (ChOx), multi-wall carbon nanotubes (MWCNTs), GNPs and poly-diallyl dimethyl ammonium chloride (PDDA) for the specific detection of choline provided an alternative, significantly sensitive, rapid and efficient approach of detection [100].

**Figure 5.**
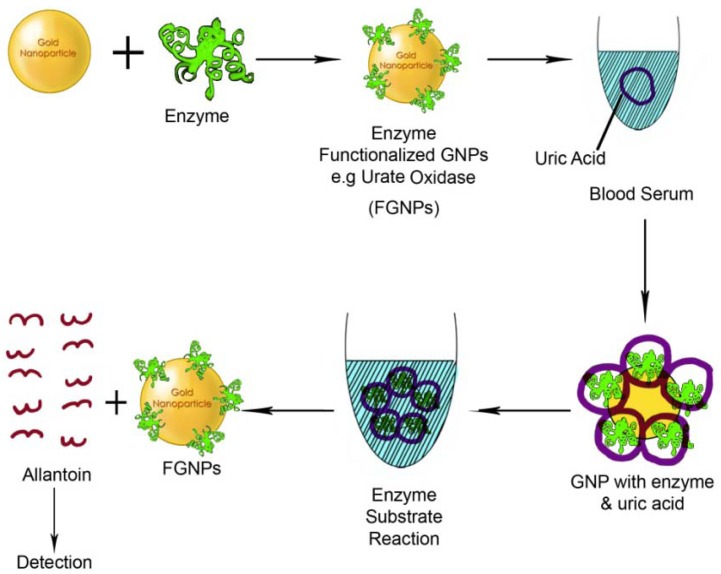
fGNP based biosensor for the detection of serum proteins.

Similarly, uric acid (UA) detection was facilitated using GNPs (Figure 5). UA is an important end product of purine metabolism abnormal levels of which are associated with various metabolic diseases such as gout, hyperuricaemia, pneumonia, kidney damage, cardiovascular diseases and Lesch-Nyhan syndrome. Several methods including colorimetric, enzymatic and electrochemical methods are available for the determination of UA concentration in human fluids. However, UA can be detected using GNPs by an amperometric method, in blood serum and urine with detection limit as low as 50 nM [101]. Correspondingly, a gold-platinum alloy nanoparticle based nanosensor with high selectivity, fast response time, sensitivity and good reproducibility was used to immobilize cholesterol oxidase on the basis of amperometric changes [102]. The principle used for the detection was based on hydrogen peroxide activity. In a yet another study, a simple but significant colorimetric biosensor was developed using gelatin-coated GNPs with 6-mercaptohexan-1-ol (MCH) for proteinase activity assay where gelatin serves as a proteinase substrate [103]. Proteinase digestion separates gelatin and brings the nanoparticles closer due to the presence of MCH, thereby causing the GNPs to aggregate and hence changing their surface plasmon resonance. The final resultant of the proteinase activity is a shift in the SPR changing the color of the solution which can be easily determined through the change in the absorbance ratio. Such method holds significant promise in the detection of proteinase activity in various biological samples.

A colorimetric “universal” biosensor was devised using ssDNA, GNPs and a water based polyelectrolyte which was found useful in the detection of DNA, proteins, small molecules, ions, *etc.* [104].

Another biosensor model was developed based on the surface plasmon resonance changes and its efficiency was tested using streptavidin [105]. Here, the absorption maximum of the scattered light by individual nanoparticles was related to the number of molecules of a given analyte bound to individual nanoparticles. The biosensor model was able to predict the molecular detection limits (minimum no. of detectable molecules) and dynamic range (maximum no. of analyte molecules bound to a nanoparticle) depending on the geometry of the nanoparticles and other parameters of the system.

### 3.4. Detection

GNPs are also being used for detection of various biological molecules including proteins, enzymes, DNA, antigens and antibodies, *etc.*

#### 3.4.1. Detection of Biological Molecules

GNPs have been used for the detection of proteins, based on their characteristic surface plasmons [106]. For this, GNPs have been functionalized using bifunctional molecules which were conjugated on one side to the GNPs through their thiol group and on the other side to the electron-rich aromatic side chains of proteins through a diazonium moiety. The model was tested using thrombin as the protein. The vibrations of the diazo-bond formed between the bifunctional molecule and the target protein tends to enhance due to the conjugation of GNPs constituting the Raman marker. After the functionalized GNPs interact with antithrombin as a sensitive recognition element, immobilized on a substrate, thrombin can be detected through surface enhance Raman Spectroscopy.

Selectively immobilized oligonucleotide modified GNPs have been used to develop a chip based array through electro-deposition on screen printed GNPs [107]. The method allows a multimodular detection based on the use of multiple oligonucleotides and also excludes the non-specific interactions. Similarly, a simple optical detection system was developed using DNA functionalized GNPs [108]. The method uses fluorescence quenching by GNPs for fluorophores attached to the detection sequences. The method is simple as it does not require the stem loop structure, characteristic of traditional molecular beacons and gives lesser background due to the electrostatic attraction between fluorescent dye and the GNPs and repulsion between GNPs and DNA. It also provides real-time monitoring, possible automation and lesser risk of contamination due to no washing steps. The reduction in fluorescence is used as a measure of binding of detection sequence with target DNA sequence. In recent study, GNPs based nanobeacons or functionalized with DNA sequences were designed for the detection of desired DNA sequences [109,110].

Similarly, GNPs have been used for detection of other biological compounds such as antioxidants which have been studied for their roles in diseases such as cancer, atherosclerosis *etc.* in suppressing the free radicals. Vitamin E (α-tocopherol) is well known for its antioxidant activity. GNPs functionalized with Trolox, an analogue of vitamin E have been synthesized using self-assembly of thiol ligand, and were evaluated for the free radical scavenging activity. The antioxidant capacity of the GNPs functionalized with Trolox was observed to be higher than that of Trolox alone, showing a promise of these antioxidant-functionalized GNPs in the treatment of various diseases [111].

Further, fGNPs have been employed for the detection of aflatoxins which are mycotoxins associated with different pathophysiological conditions in humans. Aflatoxin AFB1 is associated with cancer. GNPs functionalized with antibodies against AFB1 have been synthesized by using electro-deposition of these antibodies on cysteamine functionalized GNPs [41]. These fGNPs were found to detect AFB1 with high efficiency and less response time.

#### 3.4.2. Detection of Microorganisms

Detection of microorganisms can be achieved by several biochemical, microbiological and molecular methods. Recent advances in the field of nanotechnology have made it possible to detect microorganisms by using nanoparticles functionalized with oligonucleotides complementary to the gene tags of the microorganisms. In one such study, oligonucleotides complementary to the unique sequences of the heat shock protein 70 (HSP 70) of *Cryptosporidium parvum* was used to functionalize GNPs, which could be used to detect the oocytes of *Cryptosporidium* in a colorimetric assay, offering a simple and robust method of molecular detection [37].

GNPs were used to detect *Salmonella enteritidis* and *Listeria monocytogenes*, where GNPs deposited within the flagella and in the biofilm network [112]. Similarly, GNP–Poly(para-phenyleneethynylene) could efficiently identify both Gram positive and negative bacteria based on the differential response by each bacteria [113]. In another study, GNPs funtionalized with hairpin DNA was used to image live HEp-2 cells infected with Respiratory syncytial virus [114]. Another immunoassay based on multi-functionalized GNPs was developed by using antibodies against protein A, a cell wall protein of the bacterium *Staphylococcus aureus*, to detect it in food samples [115]. For this, gold electrode was modified by stepwise adsorption of 1, 6-hexanedithiol, GNPs and IgG and the changes in the electron transfer resistance were correlated to the deposition of functionalized GNPs. The increments in the amplified impedance showed good correlation with the protein A detection limits.

A gold nanoparticle based chemiluminescence assay was designed for the detection of *Staphylococcus* enterotoxin B (SEB) [116]. Antibody against SEB was bioconjugated to the GNPs through physical adsorption followed by adsorption of the complex on a polycarbonate surface. The SEB was then detected based on sandwich type ELISA and chemiluminescence signal arising from the secondary antibody. The method was found to be simple, easy and highly sensitive with a detection limit of ∼0.01 ng/mL.

Recent increase in the extent of antibiotic resistance in various microbial pathogens has made it necessary to design suitable methods for the detection of antibiotic resistant organisms. A simple colorimetric assay was developed using GNPs functionalized with β-lactam antibiotics [117]. Upon encounter with β-lactamase the GNPs can be made either to aggregate or disaggregate so as to give a visible color change depending upon the attached linker groups. For example, thiol group when used as linker between GNP and the antibiotic is cleaved making the GNPs disaggregate, resulting in a color change.

### 3.5. Other Applications of GNPs

#### 3.5.1. Enzyme Immobilization

GNPs have been used as immobilization matrices for enzymes. GNPs with a carboxyl terminated thiol group were functionalized through the attachment of the enzyme glucose oxidase [118]. The immobilized enzyme was found to be more stable thermally as compared to free enzyme. Such immobilized systems can be very useful in several biotechnological processes in food and environment fields. Hollow gold nanoshells entrapping horse radish peroxides have been synthesized for detection of small molecules which can enter the nanoshells [119]. This method helps the enzyme remain active in nanoshells, making it useful for various biotechnological applications.

Bi-enzyme functionalized magnetic nanoparticles were synthesized using three layer nanoparticles comprising of Fe_3_O_4_ magnetic core, a prussian blue interlayer and a gold nanoshell coupled to the enzymes hydrogen peroxide and glucose oxidase [120]. This biosensor was tested using the carcino-embryonic antigen (CEA) and α-fetoprotein (AFP) as model systems, which gives an amplified signal in terms of electrochemical activity and enzyme catalysis. These magnetic nanoparticles can be regenerated using an external magnetic field. Such a biosensor provides an extensive method of multiple detection methods with high reproducibility and sensitivity.

#### 3.5.2. Immunoassay

**Figure 6.**
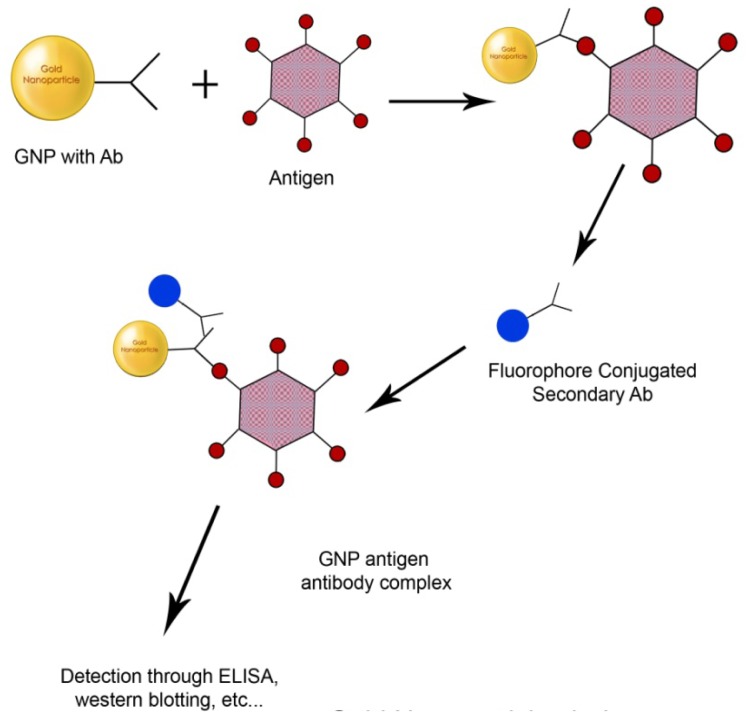
Antibody functionalized GNPs for use in immunoassay.

Various immunoassays have been designed using GNPs functionalized with antibodies such as human IgG and antibodies against pathogenic bacteria [62,121]. Immunosensors have been recently developed using single chain fragment variable recombinant antibodies (scFv) instead of traditional mono or polyclonal antibodies (Figure 6).

The scFvs are small heterodimers that are composed of the antibody variable heavy (VH) and light (VL) chains connected by a peptide linker that is used to stabilize the molecule. The scFv antibodies offer several advantages over the F_ab_, such as smaller molecular size, labeling fidelity, their designs and ability to be mutated as per the need. They also represent the smallest fraction of the antibody needed for binding to the antigen. GNPs functionalized with engineered scFv containing either a cysteine or histidine in its linker region was used to develop a colorimetric immunoassay [39]. scFv can be mutated and assembled using the phage display technique to expose specific amino acids. Mutated scFv fragments exposing the cysteine residues have been shown to form gold-thiolate bond. These were also found to adsorb on gold surface forming a monolayer giving appropriate orientation for the antigen binding. These biosensors involving GNPs of size <60 nm were found to provide sensitivity equal to or even better than the traditional fluorescence based biosensors. These scFv-cys stabilized GNPs have been shown to undergo a color change from red to purple upon addition of rabit IgG. The method was found to be highly efficient, sensitive and had a very low detection limit. Similarly, another biosensor based on engineered recombinant A10B scFv has been developed for detection of protein A as a model through self-assembled monolayer formation detected using GNPs coated with protein A with a 42 fold increase in the the detection limit as compared to A10B F_ab_ [40].

#### 3.5.3. SNP Detection

**Figure 7.**
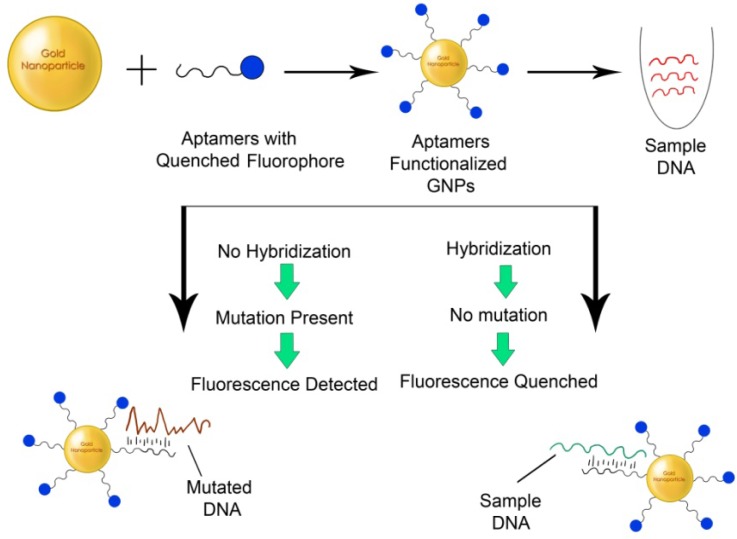
GNPs functionalized with ssDNA for Single nucleotide polymorphism (SNP) detection.

Single nucleotide polymorphisms (SNPs) have by far been the most appropriate method for the detection of point mutations or polymorphisms in various genes, which can be easily, detected using complementary single stranded DNA molecules (Figure 7).

SNPs are often associated with disease detection including diabetes mellitus, β-thalassemia, *etc.* GNPs functionalized with single-strand-specific-nucleases have been used to detect SNPs [122]. Likewise, a simple colorimetric assay was developed using DNA functionalized GNPs to detect SNPs in the human p53 gene [123]. This was successfully used to detect 12 point mutations in the human p53 gene as compared to wild type method showing a simple approach towards the detection of altered nucleotide sequences. This method neither needs complicated modification of GNPs or DNA, nor additional requirement of DNA probes, signal amplification or temperature control thus providing advantages over currently available methods.

#### 3.5.4. Metal Sensors

Development of an easy colorimetric assay to detect uranium has been achieved by using DNAzyme-GNPs system [124]. Traditionally, uranium in the environment is detected using complex biophysical techniques such as fluorimetry, ICP-MS and atomic absorption spectroscopy. However, these methods are difficult to be used on-site. DNAzyme-GNP system provides an alternative to the traditional methods. DNAzymes are catalytic DNA molecules developed *in vitro* with specific affinities to metal cofactors such as Uranyl (UO_2_^2+^) which is the most common bioavailable form of uranium. These biosensors were able to detect uranium in two ways, either by disassembly of DNAzyme functionalized GNPs in the presence of uranyl ions causing a visible color change from purple to red (“turn-on” method) or by “turn-off” method which was based on different adsorption properties of single and double stranded DNA on GNPs in the presence of uranyl ions. The method was significant as it could detect uranyl below the maximum contamination limits determined by the US environmental protection agency. GNPs functionalized with aza-crown ether acridinedione were developed as a fluorescent chemosensor for metal ions based on the shift in the surface plasmon resonance of GNPs with aggregation of nanoparticles by the sandwich complexation [125].

GNPs functionalized with L-cysteine were used for the detection of mercury (Hg^2^**^+^**). In the presence of UV light and Hg^2^**^+^**, these GNPs tend to aggregate resulting in their detection and making them useful biosensor for on-site applications [126]. Similar biosensor for Hg^2^**^+^** detection was developed using oligonucleotide fGNPs [127].

#### 3.5.5. In Microscopy

Functionalized GNPs have found their usage in electron microscopy [17]. The problem of limited resolution of Cryo-electron microscopy single particle analysis due to poor alignment of samples can be obviated by using two dimensionally arranged protein arrays labeled on GNPs through genetic tag sites on proteins. GNPs functionalized with nickel-nitrilotriacetic acid were used and *Mycobacterium tuberculosis* 20S proteasomes with 6x-histidine tags were assembled into 2D arrays and were used for three-dimensional reconstruction of biological macromolecules.

## 4. Tissue Distribution of GNPs

**Figure 8.**
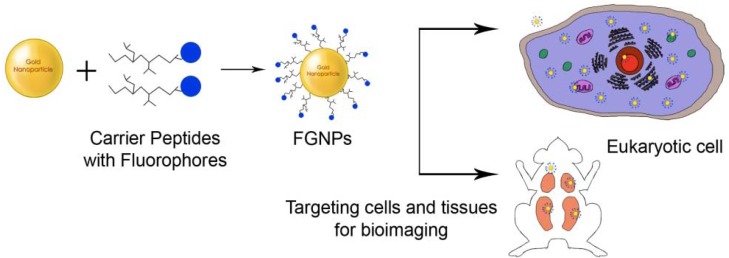
Gold nanoparticles functionalized with specific markers for Biodistribution studies.

Biodistribution studies or tissue kinetics is a method for understanding the intracellular trafficking and fate of the nanoparticles in the animal system (Figure 8).

Recently some studies have been conducted in various animal models describing the passage and clearance of the nanoparticles *in vivo*. The biodistribution of gold nanoparticles was shown to be dependent on the geometry of these nanoparticles as well as their surface chemistry.

The biodistribution has also been attributed to the type of coating or stabilizing agent used in the preparation of the gold nanoparticles as found in a study on swine [128]. The gum arabic stabilized GNPs were distributed in the liver whereas maltose stabilized GNPs were distributed in the lungs. The biodistribution actually followed a first round of distribution followed by another round of redistribution-elimination. In a more recent study, the GNPs were shown to cross the blood brain barrier and have a non-saturable deposition in the brain [129]. Also, the GNPs levels were found to decrease over time indicating efficient clearance from the body. GNPs accumulated in various organs without significant toxicity, as observed by cyto-pathological examinations in mice. Thus, promising the use of these nanoparticles to target brain and other organs. In a more detailed study, the GNPs were found to be distributed to sub-cellular targets including vesicular lysosomes/endosome like structures of the macrophages and the Kupffer cells in the liver [130]. The Kupffer cells were shown to uptake GNPs readily, indicating that they are the major cell types involved in the removal of GNPs, as evident from the decreased load of these GNPs with time. Also, GNPs based first generation anticancer drug CYT-6091 HEG-Thiol-TNF α was studied for its biodistribution and drug loading capacity [131]. The gold was found to be mostly distributed in the liver due to its size, after the release of TNF α. However the amount of residual GNPs decreased over time. The overall effect included an enhanced uptake of TNFα with lesser adverse effects.

The biodistribution of the GNPs in various tissues is also attributed to their interaction with various plasma proteins which affects their biocompatibility and therapeutic efficacy [132]. GNPs attached to tumor necrosis factor accumulate mostly in the liver and spleen and did not dissipate even after a month. Also the biodistribution of GNPs was correlated to the amounts of polyethylene glycol (PEG) used for functionalization and the injection dose [133].

Biodistribution studies on PEGylated gold nanorods and nanospheres showed their significant deposition in liver and spleen of ovarian tumor bearing mice *in vivo*. However, in other organs gold nanorods were found to accumulate more than nanospheres. Also nanorods had longer circulation times than the nanospheres [134]. The biodistribution of gold nanorods was studied on tumor-bearing mice after intravenous injection and enhanced permeability and retention. It was shown that more the amount of PEG, more is the protection of these fGNPs against reticulo-endothelial system (RES). Functionalization with a PEG: Gold molar ratio of 1.5 offers an enhanced permeability into tissues and higher retention. When more than 19.5 μg of gold was injected, the RES uptake in the liver is saturated and remaining fGNPs are distributed to liver and tumor.

In a size dependent biodistribution study of spherical GNPs, the 10 nm GNPs were more profoundly distributed in blood, liver, spleen, kidney, testis, thymus, heart, lung and brain in rats, whereas the larger particles were limited to blood, liver and spleen [135]. GNPs of size around 15 nm have been found to pass the blood brain barrier and be deposited in the brain [136]. Similarly, GNPs as big as 200 nm were able to make their way into blood, stomach and pancreas but, to a very small extent. The distribution of GNP was also observed to be shape-dependent. Similarly, GNPs of size 1.4–200 nm with negative charge on surface as well as 2.8 nm with positive charge were tested for their biodistribution behavior in rats [137]. The distribution of GNPs was found to be both size as well as charge dependent, which could also be attributed to their protein binding and exchange behavior. From the biodistribution studies on PEGylated GNPs, it was evident that they were mostly retained in the lungs, spleen and in the liver [21]. The biokinetics of these GNPs after intravenous and intra-tracheal applications of PEG modified GNPs compared to bare GNPs have been studied using radioactive-labels in rats. The PEGylated nanoparticles were found in the circulation whereas the non-PEGylated ones stayed in the liver and spleen. When introduced intra-tracheally, both types of nanoparticles were found in the lungs. In another study, of PEG-coated GNPs were used to study size dependent tissue kinetics [22]. High concentrations of 4 and 13 nm GNPs were observed in the blood for 24 h which was cleared by 7 days, whereas 100 nm GNPs were completely cleared by the end of 24 h. Also, 4 and 13 nm GNPs were detected in the mesenteric lymph nodes, and remained until 6 months, with slow elimination, whereas, 100 nm GNPs were taken up rapidly into the liver, spleen, and mesenteric lymph nodes with less elimination phase. GNPs were shown to be entrapped in cytoplasmic vesicles and lysosomes of Kupffer cells and macrophages of spleen and mesenteric lymph node. 4 and 13 nm GNPs also transiently activated phase I metabolic enzymes, in liver tissues from 24 h to 7 days, whereas 100 nm GNPs did not.

In another recent report, GNPs were rapidly and consistently accumulated in liver, spleen, kidney and testis when injected in mice [138], liver being one of the first sites of accumulation, followed by lungs, spleen, kidney and blood. The level of GNPs in lung decreases over a week's time whereas a delayed accumulation is observed in kidneys. Interestingly, no accumulation occurs in the brain. The genes affected mostly due to the GNP accumulation include those involved in detoxification, lipid metabolism, cell cycle, defense response, and circadian rhythm.

Gold composite nanodevices (CNDs) were used to study the effects of size and/or charge on the levels of selective uptake in mouse tumor models *in vivo*, by certain organs over others without any specific targeting moiety on them [139]. It was suggested that the 5 nm CNDs were more advantageous when used with the PAMAM dendrimers against the tumor cells for targeting, as compared to their larger counterparts. The explanation for this could be low immune response and organ specific recognition, making them more suitable as carriers for anticancer drugs or targeting molecules.

## 5. Toxicity of GNPs to Biological Systems

In spite of their extraordinary capacity to bioconjugate to various molecules, there have been studies showing GNPs to be cytotoxic due to their inherent physio-chemical properties. In a recent report, embryonic stem cell test (EST), was developed to check the embryotoxicity of the GNPs [140]. The EST is an *in vitro* standard assay, used to classify substances as strongly, weakly or non-embryotoxic. The embryonic stem cells (ESCs) were exposed to GNPs for 5 days to assess the cytotoxicity which follow the order: gold salt (HAuCl_4_·3H_2_O) > cobalt ferrite salt (CoFe_2_O_4_) > cobalt ferrite nanoparticles coated with silanes (Si–CoFe) > GNPs coated with hyaluronic acid (HA–Au). The ∼5 nm gold nanoparticles have been shown to induce oxidative stress and toxicity in blue mussel *Mytilus edulis*, at 750 ppb concentration after 24 h [141].

Similarly, the presence of sodium citrate residues (used as stabilizing agent for synthesis of GNPs) on the surface of GNPs elicited toxicity in alveolar cell lines *in vitro* [142]. Sodium citrate not only compromised cell viability but also affected cell proliferation. However, these nanoparticles remained localized into the membrane bound vesicles and were not freely dispersed in the cytoplasm.

Effect of polycaprolactone (PCL) coating on internalization and cytotoxicity of GNPs was evaluated on ECV-304 cells [143]. In comparison to PCL coated GNPs, bare GNPs were shown to have significant changes in the cell morphology and cytoskeleton. Also PCL coated GNPs were shown to be lesser cytotoxic as compared to bare GNPs.

The GNPs were found to induce death response in carcinoma lung cell line A549 [144]. However, BHK21 and HepG2 cell lines remained unaffected by the treatment. It was observed that the gradual increase in the GNP concentration induced a proportional cleavage of poly (ADP-Ribose) polymerase which activated caspases revealing that the effect of GNPs varies with cell type. PEGylated Raman-active GNPs (PEG-R-GNPs) comprising of an interchangeable Raman organic molecule layer held onto a gold nanocore by a silica shell have been evaluated for their cytotoxic effects on human cell lines such as HeLa and HEpG2 [145]. Addition of these fGNPs caused reactive oxygen species (ROS) generation, which was balanced by antioxidant enzyme up-regulation. An increased cellular toxicity was observed which was related to increased oxidative stress, which in turn is due to the antioxidant defenses set up by the cells in response to those ROS.

Depending upon their size and shape, the possibility of their internalization differs and so does their cytological effects. 13 nm-sized GNPs coated with PEG (MW 5000) in an *in vivo* study in mice induced acute inflammation and apoptosis in the liver [146]. The retention time for these nanoparticles in the liver and spleen was up to 7 days after injection. Moreover, the PEG-coated GNPs were also observed in the cytoplasmic vesicles and lysosomes of liver Kupffer cells and spleen macrophages. These toxic effects of GNPs have also been observed *in vivo* using mouse model [147] which was correlated to their size. GNPs with sizes of 5, 10, 50 and 100 nm were not harmful. The mice injected with these GNPs showed fatigue, loss of appetite, loss of weight, fur color change, crooked spine and finally death. Pathological changes observed included increased Kupffer cells in the liver, loss of structural integrity in the lungs, and diffusion of white pulp in the spleen. The pathological abnormality was associated with the presence of GNPs at the diseased sites. However, the toxic effects were reduced when these GNPs were functionalized with peptides, as the peptides elicited the antibody production.

### 5.1. Effect on Gene Expression

Studies have been conducted to get an insight into the genetic effects of gold nanoparticles uptake by cells *in vitro* and *in vivo*. GNPs functionalized with DNA were used as potential carriers for shRNA. These carrier gold DNA nano-conjugates were found to be efficient in the delivery of genes such as p53, Mcl-1 gene in various cell lines with a knock-down efficiency of 80–90%. Also, the efficiency of gene delivery was comparable to that of liposome mediated gene delivery [148]. In another similar study, GNPs were functionalized using thiolated, partially single stranded DNA sequence of RNA I, involved in the replication of *ColE1*-type plasmid in *Escherichia coli* [35]. The RNA I oligo acts as a cargo due to its non-complementary nature to human genes. This oligo is attached to the antisense RNA for human p53 gene and GNP, and then tested for its ability to bind to the p53 mRNA. It was found that this gene delivery system is stable for a longer period of time with no change in cellular physiology and holds promise for the efficient delivery of siRNA, ribozyme, DNAzyme, and peptide-nucleic acids. Similarly, GNPs functionalized with antisense DNA could enter the pancreatic cells with minimum toxicity and were found to regulate the transgene expression [36]. Transplantation of these functionalized GNPs treated pancreatic islets cells were found to cure diabetic nude mice showing their extensive promise as carriers for antisense DNA. In a similar study, GNPs functionalized with thiolated oligonucleotides have been used to inhibit transcription and translation *in vitro* [149]. It has been demonstrated that the resultant is a synergistic effect of GNPs and antisense sequence blocking the T7 promoter. The GNPs have been reported to have a protective effect on the oligonucleotides against degradation due to nucleases, thereby rendering them effective against the targeted genes. They have also demonstrated the use of start and stop codon complementary oligonucleotides in shutting on and/or off of genes by altering the signals needed during transcription and translation. Thus, this approach holds promise in regulating genes at both transcriptional and translational level. Another similar study evaluated the cellular response to oligonucleotide functionalized GNPs [150]. These oligonucleotide fGNPs were recognized by HeLa cells with no measureable change in the gene expression profile as opposed to GNPs with weak ligands.

Polyvalent RNA-GNP conjugates densely functionalized with synthetic RNA oligonucleotides were designed to function in the RNAi pathway [38]. These particles were synthesized to be free of degrading enzymes with high surface loading capacity for siRNA duplexes, and contained an auxiliary passivation agent for increased stability in biological media. The resultant conjugates had a half-life six times longer than free dsRNA and could readily enter cells without the use of transfection agents with a high gene knockdown capability in a cell model. Similarly, amine functionalized GNP have also been used for the intracellular delivery of siRNA with high efficiency and lesser cytotoxicity [26]. The method uses the basic principle of attachment of negatively charged siRNA-PEG complex to the positively charged GNPs. The complex is easily cleavable under the reductive cytosolic environment releasing the siRNA into the cytosol. Also the siRNA-PEG-GNP complex has been shown to inhibit the expression of a gene causing severe cytotoxicity. Thus, the ability of these fGNPs to make their ways through various intracellular organelles can be exploited to make them carry our desirable cargo to a targeted destination.

Specific gene regulation through RNAi has been reported using GNPs loaded with siRNA against ribonucleotide reductse in a study based on human clinical trial of patients with melanoma [151].

Also, GNPs functionalized with specific DNA sequences were used to analyze the expression of related genes. In a simple study, it was found that DNA-derivatized GNPs can be used to detect the expression of certain genes over the others [152]. Another study on molecular effects of GNPs by HeLa cells revealed that the GNPs did not trigger significant cytotoxicity in spite of being taken inside the cell. However the GNP could interact with intracellular components and trigger specific stresses without inducing significant changes in the expression levels of genes involved in stress response pathways [153].

GNPs have been related to the changes in the levels of expression of several genes. Gene expression changes in mice were observed in liver and spleen after a single GNPs intravenous injection [138]. The primary genes affected by GNPs included those involved in lipid metabolism and the cytochrome P450 family genes. Some of the cytochrome P450, family genes had an elevated level of gene expression whereas cytochrome P450 family 2, subfamily c, polypeptide 40 showed a reduced gene expression. The response of the gene expression to GNPs was also related to the organs being affected. However, some genes were found to be equally affected in liver and spleen, including the genes involved in DNA dependent transcription and circadian rhythm. PEG-coated GNPs (4 and 100 nm) have also been associated with significant changes in the levels of gene expression upon intravenous administration in mice. Similarly, when 4 and 100 nm particle sizes were administered intravenously to BALB/c mice, it showed significant changes in genes [22]. Almost 170 genes have been affected by the 4 nm GNPs whereas those of 100 nm size induced nearly 224 genes. Some of the genes having an altered expression belonged to cellular processes such as apoptosis, cell cycle, inflammation, and metabolic process, stress genes, signal transduction, and metabolic process.

## 6. Conclusions

In conclusion, GNPs can be considered as extraordinary molecular carriers for the targeting, intracellular trafficking and delivery of a huge array of biomolecules including DNA, RNA, proteins, peptides, drugs, genes and other molecules of therapeutic significance. They do not cause significant cytotoxicity due to their physiochemical properties. Despite these preliminary studies, efforts need to be taken for designing GNPs to enhance the bioavailability of these fGNPs with less immunogenicity and cytotoxicity to be used *in vivo.* A judicious choice between the size and functionalization method of the GNPs is a prerequisite for the use of GNPs in various biomedical applications.

## Figures and Tables

**Table 1.** Summary of common functionalization methods and their applications.

**S. No.**	**Functional Group**	**Ligands/Carrier Molecule**	**Key Feature**	**Application**	**Reference**
1	Polyethylene Glycol (PEG)	PEG with ligands such as a dye attached through thiol group	Adherence to the cell membrane	Cellular and intracellular targeting, biodistribution studies	[21–25]
2	Amine Group	PEG	siRNA carrier	Useful in RNAi technology	[26]
3	Carboxyl Group	Proteins	-	Various depending on the protein	[27]
4	Peptide	Cell surface receptors, amyloid inhibitory peptide + sweet arrow peptide, antibody, octrotide peptide	Cytoplasmic and nuclear translocation, adjuvant, targeting carcinoma cells analogue of somatostatin	Cellular and intracellular targeting,macrophage and pro-inflammatory cytokine elicitation bioimaging imaging of cancer cells	[28–32]
5	DNA	Aptamer, PEGylated gold-poly (β-amino ester), Thiolated ssDNA of RNA I gene, antisense DNA oligonucleotides	Targeting Prostate cancer cells, siRNA carrier, binds to antisense RNA of p53	Bioimaging, gene delivery rnai-regulation of transgene expression, detection of specific genes e.g., for microbial detection	[33–37]
6	RNA	Polyvalent RNA-gold nanoconjugates	-	RNAi	[38]
7	Antibodies	scFv Antibodies against various pathogens	Smaller size, label fidelity	Immunoassays treatment and diagnosis e.g., antibodies against aflatoxins	[39–41]
